# A Comparison of Nursing and Pharmacy Students’ Perceptions of an Acute Care Simulation

**DOI:** 10.3390/healthcare10040715

**Published:** 2022-04-12

**Authors:** Jill Pence, Shannon Ashe, Georges Adunlin, Jennifer Beall

**Affiliations:** 1 College of Health Sciences, Samford University, Birmingham, AL 35229, USA; jnpence@samford.edu (J.P.); sashe@samford.edu (S.A.); 2 McWhorter School of Pharmacy, Samford University, Birmingham, AL 35229, USA; gadunlin@samford.edu

**Keywords:** interprofessional, simulation, acute care, nursing, pharmacy, students, standardized patients, high fidelity, SPICE-R2

## Abstract

Patient outcomes are improved when healthcare professionals work collaboratively. In order for future professionals to have these entry-level skills, students from different disciplines must work together in scenarios simulating patient care. This paper provides an overview of a large-scale, acute care simulation involving students of different disciplines, including nursing and pharmacy. A survey using the validated Student Perceptions of Interprofessional Clinical Education Revised (SPICE-R2) tool was administered to students participating in the simulation prior to and within 1 week of the simulation. There were between-group statistically significant differences on two items on the pre-simulation survey and two items on the post-simulation survey. Student participants reported more positive perceptions after the simulation on every item except for “During their education, health professional students should be involved in teamwork with students from other health professions to understand their perspective roles”. The authors concluded that an interprofessional acute care simulation allowed students in both professions to recognize the value of a team approach to patient care.

## 1. Introduction

Providing patient-centered care and improving patient outcomes are this century’s primary focuses in medicine. Following the call to action by the Institute of Medicine (IOM) in 1999 to improve patient care through interprofessional practice and collaboration, the World Health Organization (WHO) provided a framework for action supporting this mission in 2010 [1]. Further, the Interprofessional Educational Collaborative (IPEC) was founded by the WHO to identify how to instill these important skills in future generations of healthcare professionals [2]. The objective was that integrated, well-structured, interprofessional education experiences would guide students to effectively communicate and collaborate with other health professionals after graduation. 

Studies over the past decade have been slowly showing that the pillars of interprofessional education (IPE) (values and ethics, roles and responsibilities, communication, and teamwork) are gained through IPE [3,4]. Various processes have been utilized to promote these skills, with the most evidence-based initiatives coming through simulation-based training (SBT) [4]. SBT provides learners with the opportunity to utilize the desired skills as well as to review the effectiveness of their skills and decide a direction for future actions [5]. When utilized in IPE, SBT provides a safe, clinical-like environment for students to utilize, refine, and enhance the skills necessary for effective interprofessional collaboration with learners from other health professions [6].

Current studies in simulation-based IPE typically involve two to three disciplines working in a single clinical environment, such as nursing and medical students in an emergency department [7]. Most of the studies show increases in students’ self-efficacy, understanding of roles and responsibilities, and attitudes towards working in healthcare teams [3]. Limitations of current studies of IPE are that the professions are connected through phone and electronic communications [8,9]. Uniquely, in this study, the activity permitted students from multiple disciplines to directly interact with each other in patient care. Additionally, there were multiple patient cases in a variety of interrelated scenarios concurrently to provide situational reality, an additional limitation of the current research [10,11]. The unique setting of the activity produced synchronous interdisciplinary collaboration of professional students in a large-scale scenario, similar to hospital-based clinical interactions.

There is a need to allow students from varied health profession programs to participate in a realistic, in-depth interprofessional simulation optimizing the IPEC competencies of communication, collaboration, and teamwork to achieve positive patient outcomes. Therefore, the purpose of this study was to compare nursing and pharmacy students’ perceptions of interprofessional clinical education before and after the activity.

## 2. Materials and Methods

### 2.1. Study Design and Setting

A total of 250 students from six health science programs at a small, private university in southeastern United States participated in a simulation-based IPE. The recruitment to participate in the cross-sectional study was limited to undergraduate nursing and professional pharmacy students due to profession-specific experiential education requirements. The university’s institutional review board approved this study.

### 2.2. Sample Size Determination

A total of 129 pharmacy and 140 nursing students participated in the IPE simulation. A target sample size for each group of students was calculated with the Qualtrics sample size calculator (https://www.qualtrics.com/blog/calculating-sample-size/ (accessed on 1 April 2022)), specifying a confidence level of 95% and a margin of error of 5%. Based on the criteria, ideal sample sizes of 97 pharmacy and 103 nursing students were determined adequate for the study. Attendance for the simulation was mandatory for pharmacy and nursing students as part of a required course. However, participation in the study via completion of the surveys was voluntary.

### 2.3. Interprofessional Education Experience

This activity took place in the fall of 2018 at a college of health sciences at a small, private university in southeastern United States. It involved 250 students from six health science programs: undergraduate nursing, respiratory therapy, pharmacy, physical therapy, nurse anesthesia, and social work. There were close to 50 faculty who participated as facilitators, resources for team members, and pre-debriefing facilitators. Additional personnel included manikin operators, runners, unit support personnel, and event coordinators, who were all filled by simulation personnel and volunteers from the participating programs.

The event took place over two consecutive days, which were each divided into two 4 h shifts. Each shift consisted of a 30 min pre-briefing; a 3 h patient care experience, including patient rounds; and a 30 min debriefing. Students were divided among the four shifts and then subdivided into seven teams, each covering a patient group/unit together.

The teams included students from each of the participating health profession disciplines. The patient units included four medical–surgical teams, one emergency room team, one ICU team, one labor and delivery team, one pediatric team, and one home care team. A total of 25 patient cases were written and filled by standardized patients (SPs) and high-fidelity manikins, with family members and support partners throughout. A total of 29 SPs and 5 manikins were utilized.

Each team was given a pre-assignment of watching orientation videos of the overall experience, event flow, and processes. Pre-briefing included unit orientation, team building, and reports on the patients on their respective units. Each patient case unfolded over the course of the day with a “shift change” in the middle of the day, where the morning shift reported to the evening shift, and the patient care continued. Students experienced processes such as patient admission, discharge, transfer, and medication dispensing, as well as tasks such as IV and catheter placement, physical therapy sessions, and bedside counseling. Patient charts were developed in an electronic health record that all students had access to, allowing for continuity of care regardless of patient location or team member utilization. Patient cases were scripted so that all materials would be included. If a team member wanted to do something that was not scripted, it was the role of the faculty resource member in that area to allow the student to discuss the rationale and why it may not be a priority. Test results were kept at a central location and were given based on a timed-release schedule (i.e., CT scan results = 15 min wait).

The simulation was paused midway through each shift for a patient rounds simulation. While patient rounds were facilitated by a faculty member, they were student-led, allowing the students to collaborate and develop a plan together. A structured debriefing session was held at the end of the shift, focusing on the objectives relating to the IPEC competencies previously mentioned.

### 2.4. Assessment

Students from each of the participating disciplines were asked to complete the Student Perceptions of Interprofessional Clinical Education Revised (SPICE-R2) survey prior to and within 1 week of simulation. The SPICE-R2 is a validated tool examining students’ attitudes toward interprofessional teams and the team approach to care of patients. It uses a five-point Likert scale and is composed of ten items across three factors (also known as subscales) [12]. The subscales include:

- Interprofessional teamwork and team-based practice (four items);
- Roles and responsibilities for collaborative practice (three items);
- Patient outcomes from collaborative practice (three items).

The SPICE-R2 items and factors are indicated in Table 1.

Because individual identifiers were not used for the pre- and post-simulation surveys, paired responses were not feasible. Other disciplines (physical therapy, respiratory therapy, and social work) also participated in the simulation, but there were not comparable numbers of participants from these disciplines to allow for comparison with nursing and pharmacy student responses. For that reason, the current study compared the perceptions of nursing and pharmacy students only.

### 2.5. Statistical Analysis

Individual identifiers were not used for the pre- and post-simulation assessments; therefore, there was no way to pair the responses and use tests designed for paired responses. We tested responses on the SPICE-R2 for normality with the Shapiro–Wilk test and used a histogram to identify major asymmetries, revealing non-normal distribution. An independent samples t-test was used to test for group differences (i.e., nursing vs. pharmacy) in self-reported prior experience with IPE activity on the pre- and post-test SPICE-R2 instrument. The Mann–Whitney U test was used to compare the scores on each of the SPICE-R2 items between nursing and pharmacy students. The Wilcoxon signed-rank test was used to determine whether there was a significant difference between the pre-test and post-test scores. The level of significance was alpha ≤ 0.05.

## 3. Results

### 3.1. Demographics

The participants in the study consisted of senior-level baccalaureate nursing students and third-year pharmacy students. Although there were undergraduate and graduate students, their clinical knowledge levels were similar due to program design and clinical experience. The study did not achieve the calculated sample size of 97 pharmacy and 103 nursing students.

In Table 2, prior exposure and participation in IPE and perception of the IPE simulation are reported.

A total of 134 students completed the post-IPE simulation survey. Of those, 51 were pharmacy students, and 83 were nursing students. While 49% of the pharmacy students reported previous experience with IPE, 41% of nursing students reported having no prior experience with IPE. The students’ perceptions were positive following the IPE simulation, with the vast majority of pharmacy students (96%) and nursing students (99%) indicating that the IPE simulation activity was a valuable learning experience. The vast majority of pharmacy students (90%) and nursing students (92%) indicated that they enjoyed the IPE simulation.

### 3.2. Evaluating Pre- and Post-Simulation Scores

Table 3 shows the results of the Mann–Whitney U test conducted.

Some notable pre- and post-test between-group differences were observed. In the pre-simulation, significant differences were observed between groups for two items relating to “My role within an interprofessional team is clearly defined” (Table 3, Item 2) and “Healthcare costs are reduced when patients/clients are treated by an interprofessional team” (Table 3, Item 6). On Item 2, the pre-simulation average score of pharmacy students was significantly higher (M = 4.59, SD ± 0.60) than that of nursing students (M = 4.33, SD ± 0.76), *p* = 0.023. On Item 6, the pre-simulation average score of pharmacy students was also significantly higher (M = 4.71, SD ± 0.53) than that of nursing students (M = 4.16, SD ± 0.91), *p* = 0.000. A mean score increase was noted on all survey items on the SPICE-R2 in the post-simulation. Between the two items that demonstrated significant differences in the pre-simulation, only one (“Healthcare costs are reduced when patients/clients are treated by an interprofessional team” (Table 3, Item 6)) remained significant post-simulation. Specifically, the Item 6 post-simulation score of pharmacy students remained significantly higher (M = 4.82, SD ± 0.47) than that of nursing students (M = 4.43, SD ± 0.87), *p* = 0.002. Statistically significant increases were observed on one other item (“Patient/client-centeredness increases when care is delivered by an interprofessional team” (Table 3, Item 9)), with pharmacy students scoring higher (M = 4.92, SD ± 0.27) than nursing students scored (M = 4.75, SD ± 0.57), *p* = 0.050.

As previously indicated, the SPICE-R2 instrument contains 10 items and 3 factors focused on interprofessional teamwork and team-based practice, roles and responsibilities for collaborative practice, and patient outcomes from collaborative practice. The analysis comparing the mean scores for SPICE-R2 factors pre- and post-simulation was completed using the Wilcoxon signed-rank test, and the results are presented in Table 4. Effect sizes are reported using Cohen’s d [13]. We considered values of 0.2 as small, 0.5 as medium, and 0.8 and higher as large effect sizes [13].

Among the nursing students, statistically significant increases in mean scores were noted for the roles and responsibilities for collaborative practice (M = 4.42, SD ± 0.83, *p* = 0.025) and patient outcomes from collaborative practice factors (M = 4.71, SD ± 0.60, *p* = 0.034) in the post-simulation. Among the pharmacy students, there was a statistically significant increase in mean score for the roles and responsibilities for collaborative practice factor within the SPICE-R2 instrument in the post-simulation (M = 4.54, SD ± 0.54, *p* = 0.047). Among the pharmacy students, the effect-size values for the three factors ranged from 0.24 to 0.39, indicating small effects. Among the nursing students, the effect-size values for the three factors ranged from 0.17 to 0.31, also indicating small effect sizes.

## 4. Discussion

The study results indicate that there were baseline differences observed between groups for Items 2 (“My role within an interprofessional team is clearly defined”) and 6 (“Healthcare costs are reduced when patients/clients are treated by an interprofessional team”) of the SPICE-R2 instrument. After the simulation, these differences remained for Item 6 and were also observed for Item 9 (“Patient/client-centeredness increases when care is delivered by an interprofessional team”). Scores increased in both groups between every item except Item 10 (“During their education, health professional students should be involved in teamwork with students from other health professions to understand their respective roles in the nursing student group”). There were also changes in factor scores from pre- to post-simulation experience. Significant changes were observed for factor R (roles and responsibilities for collaborative practice).

A literature search revealed two studies that were published comparing nursing and pharmacy students’ perceptions of an interprofessional simulation that used SPICE instruments [14,15]. In our study, there was an increase in mean score overall for all students as well as for individual scores from pre-test to post-test. No significant difference was seen based on discipline between pre-test and post-test. Similarly, a study of nursing and pharmacy students showed an increase in perceptions of healthcare teams following an acute care experience [14]. Fusco and Foltz-Ramos investigated the change in perceptions of interprofessional practice in nursing and pharmacy students before and after a high-fidelity simulation experience. The SPICE-R tool was used for this study. There were no decreases in median or interquartile range scores from pre-test to post-test for either discipline. Another study by Muzyk and colleagues investigated attitudes of nursing and pharmacy students in an interprofessional substance use disorder course [15]. The SPICE-R2 instrument was used, and results were reported by subscales. Similarly to our study, there were statistically significant differences between pre- and post-course surveys in the subscale of roles and responsibilities; however, the results are presented with nursing and pharmacy students combined.

Evaluating simulation-based IPE is reliant on the goals and objectives to be assessed. Currently, there are multiple tools available to measure student objectives, and all focus on some, if not all, of the tenants of IPE as described by IPEC in 2016 [3]. The SPICE-R2 instrument focuses on the student perceptions of roles and responsibilities of interprofessional groups, teamwork and team-based practice, and patient outcomes [12]. Modifications to the initial instrument were designed to enable use by professions outside of medicine and pharmacy. In previous studies, nursing and medical students improved their perceptions of interprofessional practice and role stereotypes [16]. Lockeman and colleagues developed a quasi-experimental pre-test–post-test study to explore whether a series of simulation experiences promoted changes in perceptions of IPE among medical and nursing students. The SPICE-R2 survey was used, as it was used in the current study. Utilizing the SPICE-R2 to identify the baseline of and changes in students’ understanding of roles and responsibilities, as well as teamwork and collaboration, provided insight into a healthcare team’s role in the dynamic patient care that is a hospital setting. 

Overall, most student respondents reported that this simulation activity was a valuable learning experience and that they enjoyed it. There were increases in average scores from pre- to post-simulation survey results in all items for pharmacy students and in all but one of the items for nursing students. The results of this assessment will be used for an ongoing evaluation of the simulation activity and to implement necessary changes for a more effective experience. 

Limitations of the current study include a lower than desirable response rate on the surveys, including a drop in responses from pre- to post-simulation surveys. As mentioned previously, the numbers of students from other disciplines who participated in the simulation were not compared to allow the comparison of nursing and pharmacy student responses. Because individual identifiers were not used, paired responses between pre- and post-simulation surveys were not feasible. Future research should be performed to compare perceptions of acute care simulations across various academic institutions to strengthen the current literature.

## 5. Conclusions

The interprofessional acute care simulation allowed students in both professions to recognize the value of a team approach to patient care. The simulation activity demonstrated the impact that IPE plays in ensuring that nursing and pharmacy students complete their educational training with the skills and competencies needed not only to be effective healthcare providers but also to be efficient members of a healthcare team. As academic institutions seek to bridge health disciplines, this study demonstrated an IPE activity that can help achieve this goal.

## Figures and Tables

**Table 1:** SPICE-R2 items and factors.

1.	Working with students from different disciplines enhances my education ^a^
2.	My role within an interprofessional team is clearly defined ^b^
3.	Patient/client satisfaction is improved when care is delivered by an interprofessional team ^c^
4.	Participating in educational experiences with students from different disciplines enhances my ability to work on an interprofessional team ^a^
5.	I have an understanding of the courses taken by, and training requirements of, other health professionals ^b^
6.	Healthcare costs are reduced when patients/clients are treated by an interprofessional team ^c^
7.	Health professional students from different disciplines should be educated to establish collaborative relationships with one another ^a^
8.	I understand the roles of other health professionals within an interprofessional team ^b^
9.	Patient/client-centeredness increases when care is delivered by an interprofessional team ^c^
10.	During their education, health professional students should be involved in teamwork with students from different disciplines in order to understand their respective roles ^a^

Factors: ^a^ = interprofessional teamwork and team-based practice (T); ^b^ = roles and responsibilities for collaborative practice (R); ^c^ = patient outcomes from collaborative practice (O).

**Table 2:** Prior experience with IPE (POST) and post-simulation perceptions.

Demographic Variable	Pharmacy Students (*n* = 51)	Nursing Students (*n* = 83)
Previous experience with IPE		
Yes	25 (49%)	49 (59%)
No	26 (51%)	34 (41%)
I believe this was a valuable learning experience		
Yes	49 (96%)	82 (99%)
No	2 (4%)	1 (1%)
Overall, I enjoyed the simulation		
Yes	46 (90%)	76 (92%)
No	5 (10%)	7 (8%)

**Table 3:** Comparison of between-group differences in average scores pre- and post-simulation.

	Between-Group Pre-Simulation Averages ^a^			Between-Group Post-Simulation Averages ^a^		
SPICE-R2 Items Number	Pharmacy Students (*n* = 52) Pre-Simulation Mean (SD)	Nursing Students (*n* = 136) Pre-Simulation Mean (SD)	*p*-Value	Pharmacy Students (*n* = 51) Post-Simulation Mean (SD)	Nursing Students (*n* = 83) Post-Simulation Mean (SD)	*p*-Value
1	4.71 (0.49)	4.61 (0.69)	0.634	4.84 (0.36)	4.66 (0.73)	0.167
2	4.59 (0.60)	4.33 (0.76)	0.023 *	4.64 (0.52)	4.46 (0.84)	0.375
3	4.82 (0.43)	4.75 (0.61)	0.553	4.90 (0.30)	4.79 (0.57)	0.317
4	4.73 (0.48)	4.61 (0.64)	0.309	4.78 (0.46)	4.69 (0.65)	0.606
5	4.01 (0.91)	3.88 (1.15)	0.834	4.50 (0.54)	4.21 (1.08)	0.482
6	4.71 (0.53)	4.16 (0.91)	0.000 *	4.82 (0.47)	4.43 (0.87)	0.002 *
7	4.84 (0.36)	4.77 (0.53)	0.461	4.90 (0.30)	4.83 (0.53)	0.538
8	4.36 (0.59)	4.30 (0.82)	0.842	4.60 (0.60)	4.49 (0.80)	0.536
9	4.80 (0.39)	4.69 (0.57)	0.276	4.92 (0.27)	4.75 (0.57)	0.050 *
10	4.80 (0.39)	4.76 (0.49)	0.720	4.88 (0.32)	4.74 (0.58)	0.142

* Results demonstrating statistical significance (*p* < 0.05) appear in bold.

**Table 4:** Comparison of pre- to post-test factor scores.

Factors	Pharmacy Students					Nursing Students				
	Pre (SD)-*n* = 52	Post (SD)-*n* = 51	Difference ^a^	*p*-value	D ^b^	Pre (SD)-*n* = 136	Post (SD)-*n* = 83	Difference ^a^	*p*-value	d ^b^
T	4.77 (0.39)	4.86 (0.35)	0.09	0.210	0.24	4.69 (0.51)	4.78 (0.56)	0.090	0.226	0.17
R	4.32 (0.57)	4.54 (0.54)	0.22	0.047 *	0.39	4.17(0.78)	4.42 (0.83)	0.250	0.025 *	0.31
O	4.78 (0.34)	4.90 (0.30)	0.12	0.061	0.37	4.54 (0.56)	4.71(0.60)	0.170	0.034 *	0.29

Pharmacy students (pre-*n* = 52; post-*n* = 51); nursing students (pre-*n* = 136; post-*n* = 83); ^a^ Cohen’s d (0.2 as small, 0.5 as medium, and 0.8 and higher as large); ^b^ Cohen’s d standardized effect size.SD: standard deviation. * Results demonstrating statistical significance (*p* < 0.05) appear in bold.
